# Identification of an occult recto-prostatic fistula with cystoscopy-assisted air colostogram

**DOI:** 10.1016/j.ijscr.2019.11.019

**Published:** 2019-11-19

**Authors:** Matthew P. Shaughnessy, Christine J. Park, Adam B. Hittelman, Robert A. Cowles

**Affiliations:** a Department of Surgery, Section of Pediatric Surgery at Yale University, 333 Cedar St, FMB 131, New Haven, CT, United States; b Department of Urology at Yale University, 1 Park Street, New Haven, CT, United States

**Keywords:** Anorectal malformation, Augmented-pressure distal colostogram, VACTERL, PSARP, Rectourinary fistula, Case report

## Abstract

**Introduction:**

Anorectal malformations (ARM) in newborns classically present with the absence of a normal anus or an abnormally located anus. In a male infant with a high ARM, an initial diverting colostomy is later followed by a definitive posterior sagittal anorectoplasty (PSARP). Prior to definitive surgery an augmented-pressure colostogram is performed to identify the location of the fistula between the rectum and urogenital tract. However, on occasion, the augmented-pressure colostogram fails to identify the location of the fistula tract. We present a case of ARM where augmented-pressure colostogram failed to identify a fistula tract, thus requiring an alternative diagnostic approach.

**Presentation of case:**

A newborn male presented with a high anorectal malformation and suspected rectourinary fistula on initial augmented-pressure colostogram. The patient ultimately underwent a laparoscopic assisted PSARP after cystoscopy with air colostogram identified the exact location of the fistulous connection in the prostatic urethra.

**Discussion:**

Augmented-pressure colostogram remains the gold standard in diagnosing rectourinary fistulae in cases of ARM. However, a number of alternative and adjunctive techniques have been proposed in recent years. We provide a brief review of the literature in addition to a case presentation highlighting the potential benefits of pre-operative cystoscopy-assisted air colostogram in male patients with ARM.

**Conclusion:**

Cystoscopy-assisted air colostogram via a distal mucous fistula can be utilized as an alternative diagnostic modality, especially when the augmented-pressure distal colostogram fails to identify rectourinary fistulae in high anorectal malformations.

## Highlights

• High anorectal malformations (ARM) are associated with rectourinary fistulae.
• Augmented-pressure colostogram is the gold standard in diagnosing recto-urinary fistulae in cases of ARM.
• Cystoscopic assisted air colostogram can be used as an adjunctive diagnostic to augmented-pressure colostogram.

## 1 Introduction

Anorectal malformation (ARM) affects both male and female infants with an incidence as high as 1 in 5000 live births [1]. While the exact etiology of these defects remains unclear, ARMs can be isolated or they can be associated with other congenital conditions, most notably as part of the VACTERL (vertebral, anorectal, cardiac, tracheoesophageal, renal, and limb anomalies) association [2]. ARMs present clinically with an abnormal anus or perineum, sometimes associated with a distal bowel obstruction. Following a careful examination of the perineum, a decision regarding the anatomic type of ARM can be reached and a plan for primary posterior sagittal anorectoplasty (PSARP) or diverting colostomy with delayed reconstruction is often made. In general, low defects can be treated with primary PSARP and higher defects with an initial diverting colostomy.

In males, ARMs are associated with a fistula between the rectum and the urogenital tract or perineum approximately 95% of the time, and the precise anatomic location of this fistula plays a critical role in the eventual approach for operative repair [3]. ARMs with rectoperineal fistulas are considered low and can be approached early via the perineum. Higher ARMs associated with rectourethral, rectoprostatic, or rectobladder neck fistulas are not as easily defined anatomically and are more safely approached first with a descending colostomy followed by a PSARP weeks to months later. This allows for growth of the infant but, more importantly, it allows the surgeon to delineate the exact location of the rectourinary fistula via the use of an augmented-pressure distal colostogram [3].

An augmented-pressure distal colostogram is performed by inserting a balloon-tipped catheter into the mucous fistula (distal stoma) and instilling contrast material antegrade under fluoroscopic visualization while providing traction to the balloon against the stoma, allowing hydrostatic pressure to build within the rectum [3]. Subsequently, the distal rectum and fistula will fill with contrast, demonstrating the presence and location of the rectourinary fistula. Omitting the distal colostogram increases the risk of intra-operative injury, particularly urologic injury, during the PSARP [4]. On occasion, however, the augmented-pressure distal colostogram does not clearly delineate a fistula [5]. We present a case of a high ARM with recto-prostatic fistula that could not be identified via an augmented-pressure colostogram, likely reflective of a small fistulous opening obstructed with mucous. The location of the fistula was successfully delineated by observing the urethral lumen with a cystoscope while infusing air via the mucus fistula, thus revealing the site of the fistula. This technique is a viable alternative when the augmented pressure distal colostogram does not successfully delineate the fistula. This work has been reported in line with the Surgical Case Report Guidelines (SCARE) criteria [6].

## 2 Presentation of case

### 2.1 Pregnancy and delivery

The neonate was a 2280 g male product of a suspected 34-week pregnancy, born to a 20-year-old G1P0 mother by normal spontaneous vaginal delivery. Of note, prenatal care was limited as the mother was unaware of her pregnancy until presenting to the emergency department with abdominal pain, nausea and vomiting on the day of delivery. Shortly after her presentation, ultrasound revealed intrauterine pregnancy with oligohydramnios. The mother was subsequently induced and the neonate was delivered vaginally with Apgar scores 9/9. On newborn physical exam, the neonate was noted to have an anorectal malformation.

**Fig. 1:**
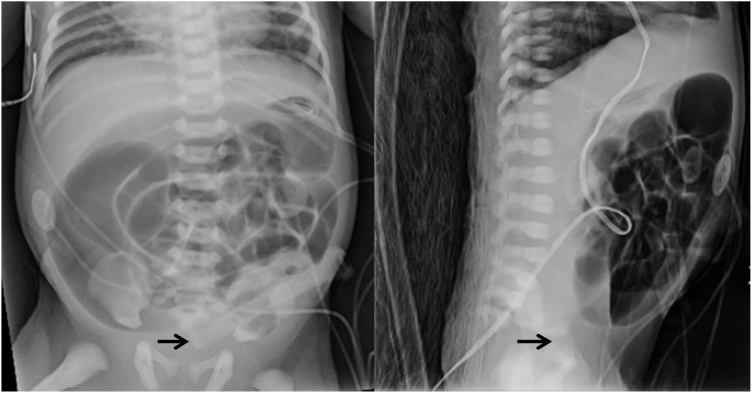
AP and lateral x-ray on day of life 0. Demonstrating passage of oral gastric tube into the gastric fundus, distended loops of bowel, and lack of air passage into the distal rectum (arrows).

Careful evaluation revealed a bifid scrotum, bilateral hydronephrosis, caudal regression syndrome with hypoplastic distal cord and sacral agenesis, atrial septal defect, and patent ductus arteriosus – consistent with a diagnosis of VACTERL association. There was no evidence of a perineal fistula on exam nor any meconium noted in the urine, and a cross table x-ray revealed no evidence of gas reaching the rectum (Fig. 1). On day of life 2, the infant underwent a diverting end colostomy with mucous fistula at the level of the proximal sigmoid colon.

### 2.2 Pre-PSARP work-up

**Fig. 2:**
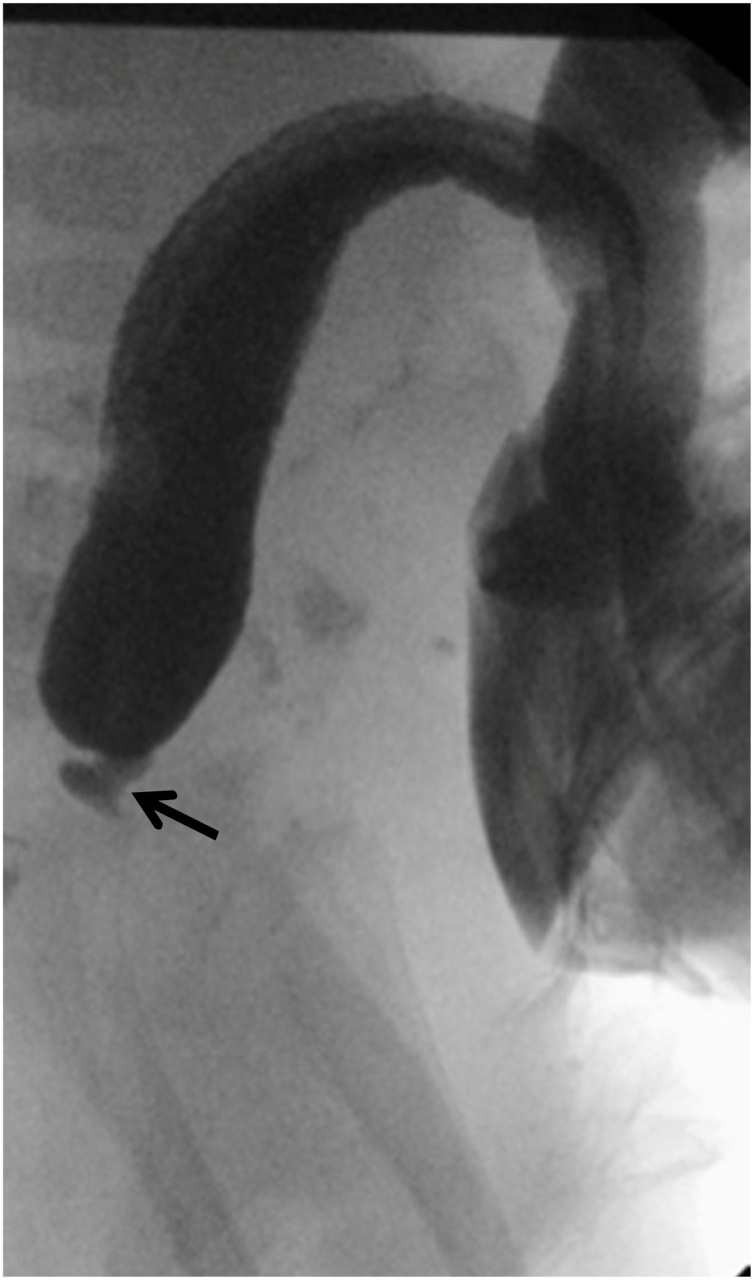
Augmented-pressure distal colostogram demonstrating normal caliber distal rectosigmoid ending with a tapered segment (arrow), however no filling of the urethra or bladder or delineation of the connection to the genitourinary tract is seen.

An augmented-pressure distal colostogram via the mucous fistula was attempted at 6 months of age (Fig. 2). The colostogram revealed a normal caliber distal recto-sigmoid, terminating approximately 4 cm from the anal dimple and faint extravasation of contrast suggestive of a fistula. However, the exact location of the fistula was not clearly delineated.

### 2.3 Localization of recto-prostatic fistula with cystoscopic assisted air colostogram

**Fig. 3:**
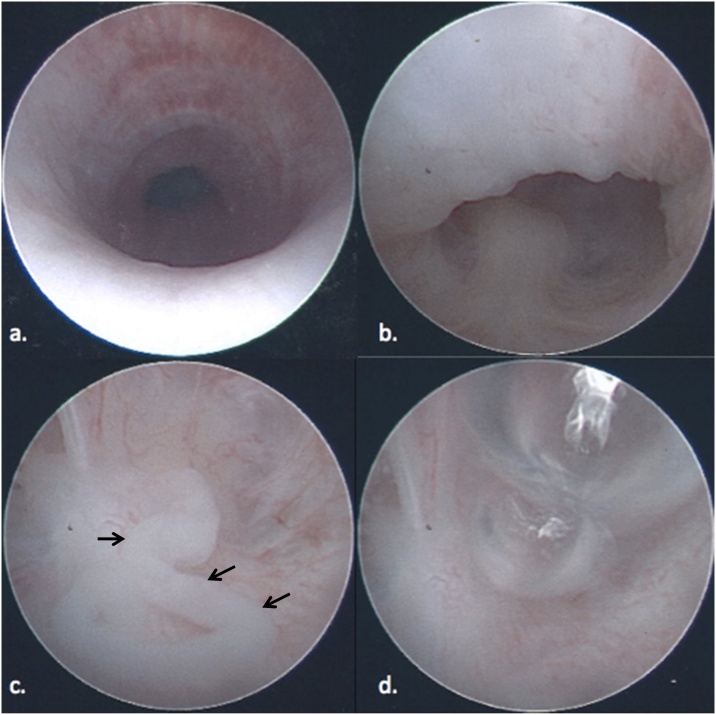
A series of cystoscopic images demonstrating the presence of mucous and air emanating from rectourethral fistula tract. (a) Normal appearing urethra, (b) verumontanum with no evidence of fistula, (c) mucous cast (arrows) expelled from fistula opening, (d) air bubbles emanating from fistula tract.

Presuming that the defect was high, a PSARP was scheduled at 9 months of age. At the outset of the procedure, cystoscopy was performed in an attempt to visualize the rectourinary fistula. A 360-degree evaluation of the entire length of the bulbar and prostatic urethra revealed no evidence of a fistula (Fig. 3a–b). The bladder was inspected and found to contain a small amount of debris, however, no obvious recto-vesicular fistula was visualized. Given the lack of visualization with cystoscopy alone, air was injected via the mucus fistula under cystoscopic visualization. A Foley catheter was introduced into the mucous fistula, the balloon was filled with saline, and gentle backward traction was applied. Air was instilled through the Foley catheter, while concurrently inspecting the urethra with the cystoscope. Shortly thereafter, a cast of mucous was expelled from an opening in the prostatic urethra (Fig. 3c), which was followed by a stream of air bubbles (Fig. 3d). This clearly demonstrated the presence and location of a recto-prostatic fistula, guiding the decision to proceed with laparoscopic mobilization and division of the fistula tract prior to a perineal approach to construct the anoplasty. An uneventful laparoscopically-assisted PSARP was then completed.

## 3 Discussion

We present a case of ARM with recto-prostatic fistula in a newborn male with VACTERL association wherein the distal colostogram was not successful in delineating the rectourinary fistula location. As a result, we describe a simple and relatively low risk diagnostic adjunct to the distal colostogram, which should be considered a viable alternative when colostogram fails to localize fistulae. Since its first description in 1991 by Gross et al., the augmented-pressure colostogram has remained a standard part of the evaluation of ARMs [7]. Colostogram allows for localization of associated fistulae in cases of ARM, classifying fistulae as either high or low in relation to the perineum. This classification subsequently guides operative approach when planning a definitive PSARP. However, even when a colostogram is performed in the hands of a skilled radiologist, fistulae can go unnoticed or remain poorly defined. As a result, many centers have proposed adjunctive strategies to assist in delineating fistula anatomy.

Since the location of the fistula is so critical to operative planning, other authors have proposed strategies for defining this anatomy. Stenstrom et al. describe a case of an endoscopically placed recto-urethral guidewire [8]. In this case, a guide-wire was passed under endoscopic guidance into a well visualized fistula opening in the distal end of the rectum and out through the urethra, acting as an “artificial benchmark” to guide surgical dissection. Koga et al. report a case series of 5 male patients where they utilize laparoscopy and cystoscopy in conjunction to measure the length of recto-urethral fistulae in ARM patients, arguing that knowledge of fistula length helps to prevent genitourinary injury [9]. Furthermore, Soccorso et al. describe the use of micturating cystography and a “double urethral catheter technique” to further characterize ARM fistulae prior to surgical intervention [10]. In resource limited institutions, particularly in Nigeria, there have been successful attempts at performing non-fluoroscopic pressure colostography by obtaining two oblique radiographs as demonstrated in a 12-patient case series by Abdulkadir et al. [11] Finally, Thomeer et al. compared MRI to colostography/fistulography in 33 neonates (22 boys), demonstrating potentially higher accuracy of MRI in diagnosing ARM with fistula without the risk of bowel perforation [12].

However, despite these reports, the literature is limited in describing viable alternatives for fistula localization when radiographic visualization of the fistula is indeterminate. The case described in this report adds to the existing adjunctive strategies for identifying and describing ARM fistulae. We describe a simple technique using common general and pediatric surgical principles that are applied in a relatively novel way and only require standard equipment found in most pediatric facilities. Similarly to the commonly utilized air leak test of a gastrointestinal anastomosis, we describe a method in which air is passed through the lumen of the rectum in order to facilitate the detection of air bubbles entering the bladder or urethra in cases suspected of having a fistula. As cystoscopy requires constant flow of water, air bubbles are easily identified passing through a fistula tract into a fluid filled bladder or urethra. This is by no means a complicated concept, but we delineate the steps as follows: 1) Pre-operative cystoscopic evaluation with 360-degree inspection of the length of the urethra and bladder and identification of the ureters; 2) Insertion of a Foley catheter into the mucous fistula; 3) Instillation of saline into the Foley balloon and application of gentle traction against the stoma; 4) Instillation of air into the rectum via the Foley catheter while visualizing the urethra with cystoscopy (alternatively, dye can be used); 5) Identification of an “air leak” via the fistula tract. It is important to note that this technique must be performed with caution to avoid bowel perforation, as is the case with augmented-pressure colostogram. Additionally, and as discussed by Stenstrom et al., an endoscope can be used as opposed to a Foley catheter to provide visualization in addition to air insufflation.

Our reported technique has limitations. Case reports knowingly lack an appropriate number of subjects to truly evaluate the described technique, warranting future studies. However, we feel that our anecdotal experience may assist other pediatric surgeons when faced with a seemingly occult ARM fistula. In this case, surgical decision making was directly influenced by the findings of cystoscopy, and we anticipate applying this technique to future cases. It is important to note that a small subset of patients (approximately 5%) have an ARM without fistula [13], particularly in the case of trisomy 21, in which up to 95% of patients with ARM have no fistulae [3]. These are often unique defects with long common channels between the rectum and urethra, thus challenging the surgeon even without fistulae. Though we do not present a case of ARM without fistula, we believe that our technique would be beneficial in these patients prior to any surgical intervention, given its relative ease and seemingly low risk profile. Furthermore, we do not intend to deter pediatric surgeons from pursuing the standard augmented-pressure colostogram pre-operatively. Augmented-pressure colostogram remains the gold standard and should be used in conjunction with any other technique as needed. However, when the colostogram cannot provide the location of a fistula in ARMs, or its findings are equivocal, we recommend cystoscopy-assisted air colostogram

## 4 Conclusion

Cystoscopy-assisted air colostogram via a distal mucous fistula can be utilized as an adjunctive diagnostic to augmented-pressure distal colostograms for localizing rectourinary fistulae in anorectal malformations.

## Funding

This research did not receive any specific grant from funding agencies in the public, commercial, or not-for-profit sectors.

## Ethical approval

As per local institutional report guidelines: a single case report does not qualify as a clinical investigation, “research,” or involve “human subjects” as defined in the federal regulations and University policy and as such a case report is not mandated to be subject to approval and oversight by the IRB.

## Consent

“Written informed consent was obtained from the patient’s guardian for publication of this case report and accompanying images. A copy of the written consent is available for review by the Editor-in-Chief of this journal on request”

## Registration of research studies

Not registered.

## Guarantor

Matthew Shaughnessy, Robert Cowles.

## Provenance and peer review

Not commissioned, externally peer-reviewed.

## CRediT authorship contribution statement

**Matthew P. Shaughnessy:** Writing - original draft, Writing - review & editing, Conceptualization, Investigation. **Christine J. Park:** Writing - original draft, Writing - review & editing, Investigation. **Adam B. Hittelman:** Methodology, Writing - review & editing. **Robert A. Cowles:** Conceptualization, Methodology, Writing - original draft, Writing - review & editing.

## Declaration of Competing Interest

None.
